# Rheopheresis for Digital Ulcers and Raynaud's Phenomenon in Systemic Sclerosis Refractory to Conventional Treatments

**DOI:** 10.3389/fmed.2019.00208

**Published:** 2019-09-18

**Authors:** Peter Korsten, Gerhard A. Müller, Jan-Gerd Rademacher, Michael Zeisberg, Björn Tampe

**Affiliations:** Department of Nephrology and Rheumatology, University Medical Center Göttingen, Göttingen, Germany

**Keywords:** systemic sclerosis, extracorporeal circulation, raynaud phenomenon, skin ulcers, plasmapheresis

## Abstract

Raynaud's phenomenon (RP) is almost universally present in patients with Systemic Sclerosis (SSc). RP represents a generalized vasculopathy and potentially lead to digital ulcers (DU), which may be complicated by superinfection, tissue necrosis, and limb loss. We report the analysis of an extracorporeal procedure in a 36-year-old female patient with diffuse SSc with refractory RP and DU despite treatment with diltiazem, candesartan, sildenafil, and intravenous iloprost. We performed rheopheresis (RheoP), a variant of double-filtration plasmapheresis, as a potential new treatment option for refractory patients despite optimal medical therapy. We performed two RheoP per week every 4 weeks for a total of 3 months. Clinical improvement in DU healing occurred with no adverse events directly related to the treatment. While there was no reduction in the number of Raynaud attacks with RheoP, a significant reduction of the duration of attacks from a median of 15 (5–45, 95% CI 10–15) to 7 (3–30, 95% CI 6–10) minutes with an improvement of the Raynaud Condition Score (RCS) improved from 4 to 2. In conclusion, RheoP is a feasible and potentially beneficial treatment modality in patients with refractory RP and DU. We propose that RheoP should be investigated in a larger number of patients in a clinical trial setting.

## Introduction

Systemic sclerosis (SSc) is a chronic autoimmune disease characterized by the presence of skin and organ fibrosis, and vasculopathy (1). Raynaud's phenomenon (RP) is almost universally present in patients with SSc (1) and is the most bothersome symptom for many patients interfering with daily activities. RP may lead to critical ischemia and the development of digital ulcers (DU), potentially leading to superinfection, necrosis, and amputation (2).

Treatment recommendations include conservative measures (e.g., avoidance of cold exposure, stress reduction, and others) as well as medical therapy (3). The latter includes antihypertensives, such as calcium-channel blockers (CCB) or sartanes, and vasodilating agents, such as intravenous iloprost (3). In Germany, bosentan, an endothelin receptor antagonist (ERA), is approved for the prevention of new digital ulcers based on randomized controlled clinical trials (4). Other agents, such as macitentan (ERA) or sildenafil [a phosphodiesterase 5-inhibitor (PDE-5i)], have not reached their primary endpoints in clinical trials (5). Overall, the evidence for the efficacy of medical therapy is limited. Therefore, new treatment options for RP and DU are required.

Vasculopathy in SSc patients may be a result of abnormal blood rheology (6). Recently, we reported differences in whole blood viscosity (WBV) in a limited number of patients with DU due to SSc (7). Therefore, modulation of WBV by extracorporeal treatment procedures is of potential interest for the treatment of RP and DU in SSc.

In this regard, there are different possible modalities which may be of potential use in SSc. Total plasma exchange (TPE) is an apheresis procedure, where the patient's blood is filtered and replaced, usually by albumin or fresh frozen plasma (FFP). The removal and replacement with this procedure is relatively unselective. Its use has been reported in a number of small scale studies (8). Their discussion is beyond the scope of this manuscript.

Rheopheresis (RheoP), by contrast, is a double-filtration plasmapheresis. In RheoP, the patient's blood is filtered through a first filter, which separates the plasma from cellular components of the blood; then, blood is filtered through the second filter (the rheofilter), which selectively removes large plasma proteins. With this modality, no replacement fluid is required, thus reducing the risk of anaphylaxis associated with FFP or albumin replacement. RheoP which has been used with success in other conditions with microcirculatory alterations, such as age-related macular degeneration (AMD), diabetic foot syndrome, and critical limb ischemia, among others (9–11). RheoP offers the potential advantage of positively influencing blood viscosity by removal of large plasma proteins, such as fibrinogen, which contribute critically to WBV. RheoP has been reported in SSc in a case report including two cases with severe DU (12). In this report, however, the effects on RP were not reported. Here, we report the effect of RheoP in diffuse systemic Sclerosis (dSSc) on DU and RP in a patient refractory to other topical and systemic treatments.

## Materials and Methods

### Description of the Index Case

The patient, a 36-year-old woman, had received the diagnosis of Systemic Sclerosis 16 years before the first presentation to our center and had been treated with intravenous iloprost in 6–8 week intervals at a different center before she attended our clinic. She had no other known illnesses. Her medication included diltiazem, candesartan, low-dose prednisolone, hydroxychloroquine, mycophenolate mofetil, sildenafil, and pantoprazole. Previous bosentan treatment had to be stopped due to severe hepatic dysfunction even after dose reduction.

Clinical examination revealed diffuse skin thickening, microstomia, and the presence of DU. DU were present on the dorsal aspects of second, third, and fifth finger of the left hand and on the first, third, fourth, and fifth finger of the right hand (one DU each). There were only minimal changes present on the finger tips, which were not classified as digital ulcers as per the current definition of digital ulcers proposed by the recent World Scleroderma Foundation (13). Laboratory investigations showed anemia of chronic disease, positive anti-nuclear antibodies (ANA), and positive anti-topoisomerase I (anti-Scl70) antibodies (abs). The remainder of the investigations, including liver function tests, and creatinine with estimated glomerular filtration rate were normal, as was urinalysis. Additional antibodies, including rheumatoid factor, anti-CCP-abs and ANCA could not be detected. Investigations for organ manifestations demonstrated gastrointestinal involvement with dysphagia and abnormalities on esophageal barium swallowing. Pulmonary function tests showed normal results, and computed chest tomography revealed subclinical basal fibrosis of both lungs affecting <20% of the lung parenchyma. Applying the DETECT algorithm (14) for the recognition of pulmonary arterial hypertension (PAH) yielded a negative result. In addition, transthoracic echocardiography did not show findings suggestive of PAH, either. Nailfold video capillaroscopy revealed a “late pattern” with widespread loss of capillaries, no hemorrhages, and no megacapillaries. A clinically significant arterial stiffness was unlikely in light of normal carotid-femoral pulse wave velocity of 5.1 m/s (values <10 m/s are considered not to be associated with macrovascular disease), and the absence of important risk factors [smoking, hyperlipidemia (LDL <100 mg/dL), no history of myocardial infarction or stroke].

We continued intravenous treatment at 6 weeks intervals for 6 months, but the patient continued to suffer from severe RP and developed new DU. Therefore, we discussed other therapeutic options. We decided to perform RheoP treatments as an exploratory treatment for refractory RP and DU.

### Protocol for Rheopheresis Treatment

RheoP was performed using a Plasauto Sigma™ dialysis apparatus (Asahi, Kasei Kuraray Medical Co., Tokyo, Japan) and the Rheofilter® ER-4000 (Asahi, Kasei Kuraray Medical Co., Tokyo, Japan). Before treatment, the patient was hospitalized, and a standard triple-lumen hemodialysis catheter (Joline GmbH & Co. KG, Hechingen, Germany) was inserted into the right internal jugular vein using standard precautions. The target was to treat 100% of the patient's plasma volume, which was estimated using the formula 40 mL × kg (body weight). All filters and tubing systems were only used once according to the manufacturer's instructions.

Two RheoP treatments per week were performed for a total number of six treatments over 3 months (two treatments per week every 4 weeks). To ensure the technical quality of RheoP, we measured the relative change of plasma proteins [fibrinogen, LDL cholesterol, immunoglobulin (Ig) G and M, and albumin] after treatment.

Other protocols for TPE based on single cases with 4 weekly treatments (15) or small studies with ten to 12 weekly treatments (9–11) have been proposed. For logistic and practical reasons, we decided to use the protocol described above, which was acceptable for the patient and could be implemented easily into practice.

### Outcome Measures and Assessments

Clinically, we visually assessed healing of present DU and the development of new DU monthly and at 3 months of follow-up. Also, we instructed the patient to record the daily number of attacks, the duration of attacks and the Raynaud Condition Score (ranging from 0 to 10, with 10 representing the most severe score) in the week before and after treatment. Kruskal-Wallis test and Dunn's *post-hoc* test for multiple comparisons were performed.

## Results

**Figure 1:**
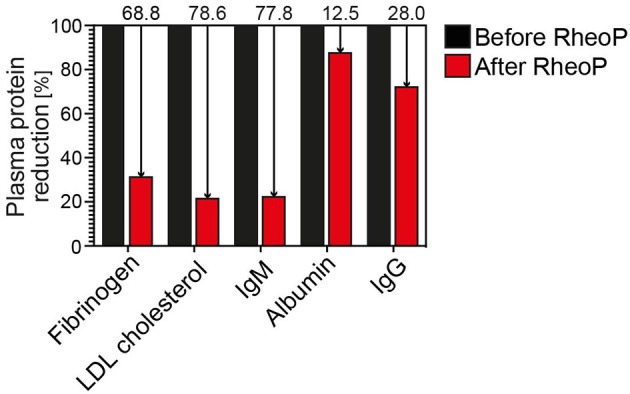
Reduction of plasma proteins before and after treatment. There is a marked relative reduction of the larger plasma proteins fibrinogen, LDL cholesterol and, IgM, whereas albumin and IgG are reduced less prominently due to their smaller molecular size. Ig, immunoglobulin; LDL, low-density lipoprotein.

The patient reported no adverse effects directly related to the RheoP except for mild discomfort associated with catheter placement. Overall, she reported reduced arthralgia, myalgias, and fatigue. Measurement of plasma proteins showed a noticeable reduction of fibrinogen, LDL cholesterol, and IgM, but only a small reduction of albumin and IgG as expected and comparable to the manufacturer's data (Figure 1).

**Figure 2:**
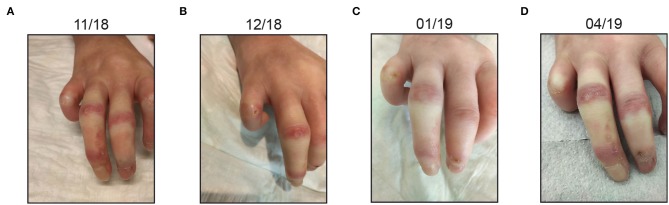
Clinical course of the patient **(A–D)**. Most prominent digital ulcers are depicted. After the last treatment, there is a near-complete healing of ulcers. Also, no ulcers developed *de novo*.

Clinically, we observed a near-complete healing of DU of the left hand, one persistent DU on the third finger of the right hand and no new DU during treatment and 3 months after the last treatment (Figures 2A–D). Of note, DU on the extensor surface of the fingers are less likely to be caused by vasculopathy alone but may be a result of skin tightening. Since there were no DU on the distal fingertips as per the current definition, the effect on DU healing was not pronounced.

**Figure 3:**
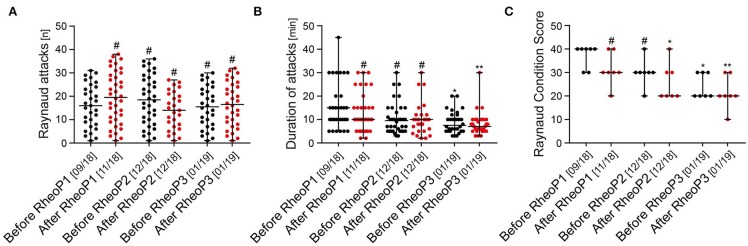
Effect of rheopheresis on Raynaud attacks and Raynaud Condition Score. **(A)** No difference was observed in the total number of Raynaud attacks (scatter dot plots with median and range are shown). **(B)** A statistically significant reduction of the median duration of Raynaud attacks was observed after the second treatment cycle and before the third treatment cycle (scatter dot plots with median and range are shown, ^#^not significant, *p < 0.05, **p < 0.01). **(C)** Raynaud Condition Score improved from 4 at baseline to 2 after the second treatment cycle (scatter dot plots with median and range are shown, ^#^not significant, *p < 0.05, **p < 0.01). RheoP, rheopheresis.

Overall, there was no reduction in the total number of Raynaud attacks during the treatment period with a median number of 16 (1–31, 95% CI 10–22) before the treatment and 16.5 (1–32, 95% CI 10–23; not statistically significant) during the week after the last treatment (Figure 3A). The patient experienced a marked reduction in the duration of Raynaud attacks when comparing before with after treatment with a reduction from a median of 15 (5–45, 95% CI 10–15) to 7 (3–30, 95% CI 6–10; ^*^p < 0.05, ^**^p < 0.01) minutes (Figure 3B). Also, the RCS showed a statistically significant improvement in the week after the second treatment cycle. RCS improved from a median score of 4 (3–4, 95% CI 3–4) to a median score of 2 (1–3, 95% CI 1–3, ^*^p < 0.05, ^**^p < 0.01; Figure 3C).

**Figure 4:**
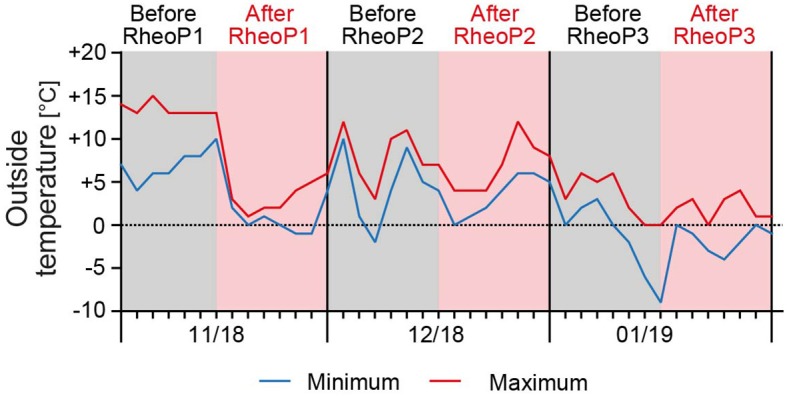
Outside temperature during the treatment period. The minimum and maximum temperatures were recorded for the patients' residential area. Temperatures ranged from −1 to 15°C in November 2018 and gradually declined to −10 to 6°C in January 2019. Data were obtained from https://www.timeanddate.de/wetter/deutschland/kassel/rueckblick (accessed on April 28th, 2019) for the respective weeks before and after treatment. RheoP, rheopheresis.

To exclude a seasonal effect of RP and DU related to the outside temperature, we recorded the minimum and maximum temperatures during the treatment period in the week before and after treatment. Temperatures during treatments were omitted because the patient was hospitalized in the respective weeks. In the first treatment month, temperatures ranged from −1 to 15°C and then gradually declined to a minimum of −10 to 6°C in the third month (Figure 4).

## Discussion

We report that RheoP is a potentially useful treatment modality in refractory DU and RP. We observed no effect on the total number of Raynaud attacks, but a statistically significant reduction of the duration of Raynaud attacks and improvement of the RCS. Also, the observed DU showed proper healing. The treatment was well-tolerated by the patient, and she reported subjective improvement of her overall disease manifestations.

TPE has been reported previously as a treatment modality, and a recent review summarized the available evidence, which is mainly based on trials with few participants and case reports (8). The treatment modalities and regimens differed markedly. Therefore, the optimal treatment frequency and duration is currently unknown. In current recommendations and expert reviews, TPE is not reported as a therapeutic modality (3, 16). This may be because TPE is not readily available at most centers and practitioners may be reluctant to consider it due to its invasiveness, sometimes requiring central venous access when peripheral access cannot readily be obtained. Compared to TPE alone, RheoP theoretically leads to an improved reduction of large plasma proteins which are relevant to blood rheology. Comparative trials, however, have not been performed. RheoP is able and specifically designed to remove large plasma proteins, including LDL cholesterol. Therefore, clinical improvements may also be due to improvement of macrovascular disease, such as PAD. The exact mechanism of action of RheoP on WBV has not been determined as of yet. We assume that improvements of blood rheology are due to the reduction of plasma proteins (as shown in Figure 1). To better understand the underlying pathophysiologic mechanisms, it would be of interest to assess WBV, either by calculating it with established formulas, which are based on the hematocrit and total serum protein concentration (17) or measuring it directly with a viscometer (7). Another potential, yet rather unlikely mechanism is the possible reduction of antibodies by RheoP. In SSc, functional antibodies against the angiotensin II type 1 receptor (AT1R) and the endothelin-1 type A receptor (ETAR) have been described (18). However, one study on immunoadsorption (IA) in SSc (NCT 01410903) has been terminated early due to slow recruitment. Also, it has been argued that antibodies recur quickly after removal by IA or other apheresis techniques (19). Thus, antibody reduction by RheoP seems to be less likely the responsible mechanism of improvement.

Our report is limited by the single individual treated and is, therefore, not generalizable to a larger group of patients. Besides, some patients may find the necessity of vascular access bothersome compared to medical therapy. However, in our experience of many patients performing weekly lipid apheresis for familial hypercholesterolemia, this is not an obstacle in clinical practice. For chronic treatment purposes, an arterio-venous fistula is a feasible option also in SSc patients. Another limitation of our report is the fact that the disease in the treated patient has already progressed severely. For example, DU were more pronounced on the extensor surface of the fingers, which are less likely to be of vasculopathic origin alone. Therefore, the effect on DU healing was not very pronounced. Nevertheless, even in this very advanced case there was an observable effect on DU healing although the effect of RheoP on RP intensity and attack duration was more intense.

In conclusion, RheoP could be a valid option for refractory DU/RP in SSc. Therefore, we plan to conduct a prospective trial comparing different treatment modalities and regimens to optimize treatment for the most severely affected patients with SSc despite optimal medical therapy.

## Data Availability Statement

All relevant data is contained within the manuscript.

## Ethics Statement

Rheopheresis was performed as part of clinical care of the patient. The patient provided written informed consent for all performed procedures and for the reporting of this case in accordance with the Declaration of Helsinki. The study protocol was approved by the ethics committee of the University Medical Center Göttingen (protocol number 32/7/19).

## Author Contributions

PK conceived the study, cared for the patient, collected and analyzed data, drafted the figures and wrote the manuscript. GM analyzed data and critically revised the manuscript. J-GR cared for the patient, collected and analyzed data and revised the manuscript. MZ revised the manuscript and assisted with data analysis and interpretation. BT drafted the figures, co-wrote the manuscript, and analyzed data. All authors contributed to manuscript revision, read and approved the manuscript.

### Conflict of Interest

The authors declare that the research was conducted in the absence of any commercial or financial relationships that could be construed as a potential conflict of interest.
